# Investigating the impact of elevated urinary trace elements on non-alcoholic fatty liver disease using vibration-controlled transient elastography

**DOI:** 10.3389/fendo.2024.1310044

**Published:** 2024-03-12

**Authors:** Chenxiao Wang, Xin Shang, Yu Fu, Panpan Guo, Ping Wang, Shuxun Yan

**Affiliations:** ^1^ Department of Gastroenterology, The First Affiliated Hospital of Henan University of Chinese Medicine, Zhengzhou, China; ^2^ Department of Endocrinology, The First Affiliated Hospital of Henan University of Chinese Medicine, Zhengzhou, China

**Keywords:** trace elements, urine, non-alcoholic fatty liver disease, VCTE, dose-response relationship

## Abstract

**Introduction:**

Non-alcoholic fatty liver disease (NAFLD) is a global public health concern. However, limited data are available on urinary trace elements and NAFLD caused by various exposure factors. This study aimed to investigate the relationship between the presence of 16 trace elements in urine and NAFLD using data from the National Health and Nutrition Examination Survey (NHANES).

**Methods:**

By utilizing the NHANES data from 2017 to 2018, 1613 participants who fulfilled the research criteria were identified from the initial pool of 2979 participants with available urine trace element detection data. Among them, 706 individuals had been diagnosed with NAFLD based on a coefficient of attenuation parameter (CAP) value of at least 274 db/m, determined using vibration-controlled transient elastography (VCTE); whereas the remaining 907 participants were classified as non-NAFLD. The data obtained were used to construct univariate and multivariate logistic regression models and restricted cubic spline models (RCS) analyses.

**Results:**

The presence of arsenic, iodine, barium, cesium, molybdenum, lead, tin, and tungsten in the urine of individuals with NAFLD showed a positive correlation with the likelihood of developing NAFLD. The risk of NAFLD had a non-linear dose-dependent relationship with urinary iodine, molybdenum, barium, and cesium. NAFLD was also associated with elevated levels of barium and cesium in urine, which were identified as significant risk factors.

**Conclusion:**

These findings suggest a positive association between exposure to trace elements in the urine and the risk of NAFLD. Specifically, urinary barium and cesium appeared to have the greatest impact on the risk of NAFLD. These results provide novel insights into the diagnosis and treatment of NAFLD.

## 1 Introduction

Excessive accumulation and degeneration of fat in liver cells, known as fatty liver disease (FLD), encompasses both alcoholic fatty liver disease (AFLD) and non-alcoholic fatty liver disease (NAFLD). Insulin resistance and genetic susceptibility are closely associated with the development of NAFLD. NAFLD primarily encompasses various forms of fatty liver disease except alcoholic fatty liver disease. This includes steatosis with or without mild inflammation (non-alcoholic fatty liver), non-alcoholic steatohepatitis, and associated cirrhosis and liver cancer (1). With increasing changes in lifestyle and eating habits, the incidence of NAFLD is increasing. Approximately 25% of adults worldwide are affected by NAFLD (2). By the end of 2020, 100 million people worldwide were affected, which greatly impacted social health care and the economy, and NAFLD has become a global problem.

Previous studies have linked trace elements to certain conditions, such as diabetes, dyslipidemia, all-cause mortality, cardiovascular disease, and cancer-related deaths (3, 4). Increasing epidemiological evidence indicates that the accumulation of metals in the body poses a potential threat to various metabolic abnormalities, thereby elevating the chances of developing obesity, liver damage, and NAFLD (5–7). Certain elements, such as lead, mercury, and cadmium, are associated with liver steatosis and fibrosis to different extents (8), with a more pronounced impact observed in females (9). Moreover, a majority of previous studies have employed the steatosis serology score, hepatic steatosis index (HSI), or fatty liver index (FLI) to detect hepatic steatosis. Consequently, despite the potential inclusion of additional research samples (10), the diagnosis of NAFLD necessitates imaging or histological examination. Approximately 78% of NAFLD patients could have liver enzyme levels within the normal range; therefore, relying on the FLI for diagnosing NAFLD may be insufficient, potentially resulting in an underestimation of NAFLD prevalence (11). Considering the intrusive nature of liver biopsy, VCTE is currently advised in the NAFLD clinical practice guidelines (12) for the non-invasive detection of liver steatosis and fibrosis, which can measure liver fat through coefficient of attenuation parameter (CAP) values. Previous studies lack VCTE as a diagnostic criterion to explore the correlation between NAFLD and urinary trace elements, especially the dose-response relationship. Hence, it is imperative to employ more precise diagnostic standards for NAFLD to facilitate future investigations.

To investigate the precise physiological functions of additional trace elements in patients with NAFLD, this study focused on defining NAFLD using ultrasound diagnosis with improved diagnostic precision. Additionally, 16 different types of urinary trace elements were focused on in this study. American adults who took part in the 2017–2018 National Health and Nutrition Examination Survey (NHANES) were the subjects of this study, and statistical analysis was performed to construct both univariate and multivariate logistic regression models, with the aim to assess the correlation between trace elements in urine and NAFLD. A restricted cubic spline (RCS) model was utilized to capture the nonlinear association between trace elements in urine and NAFLD, as well as to investigate the dose-response relationship with the risk of NAFLD to offer guidance for preventing NAFLD and identifying its underlying factors.

## 2 Materials and methods

### 2.1 Subjects

Data were obtained from the NHANES, which evaluated the health and nutrition status of adults and children in the United States. The National Center for Health Statistics (NCHS) (13) conducted the survey. Each participant was provided with extensive measurements and standardized interview questionnaires, and the survey adhered to the applicable guidelines and regulations. All participants provided written consent prior to the study. Previous literature (14) has published extensive information on NHANES, which can be accessed at the following URL: https://wwwn.cdc.gov/Nchs/Nhanes/.

**Figure 1:**
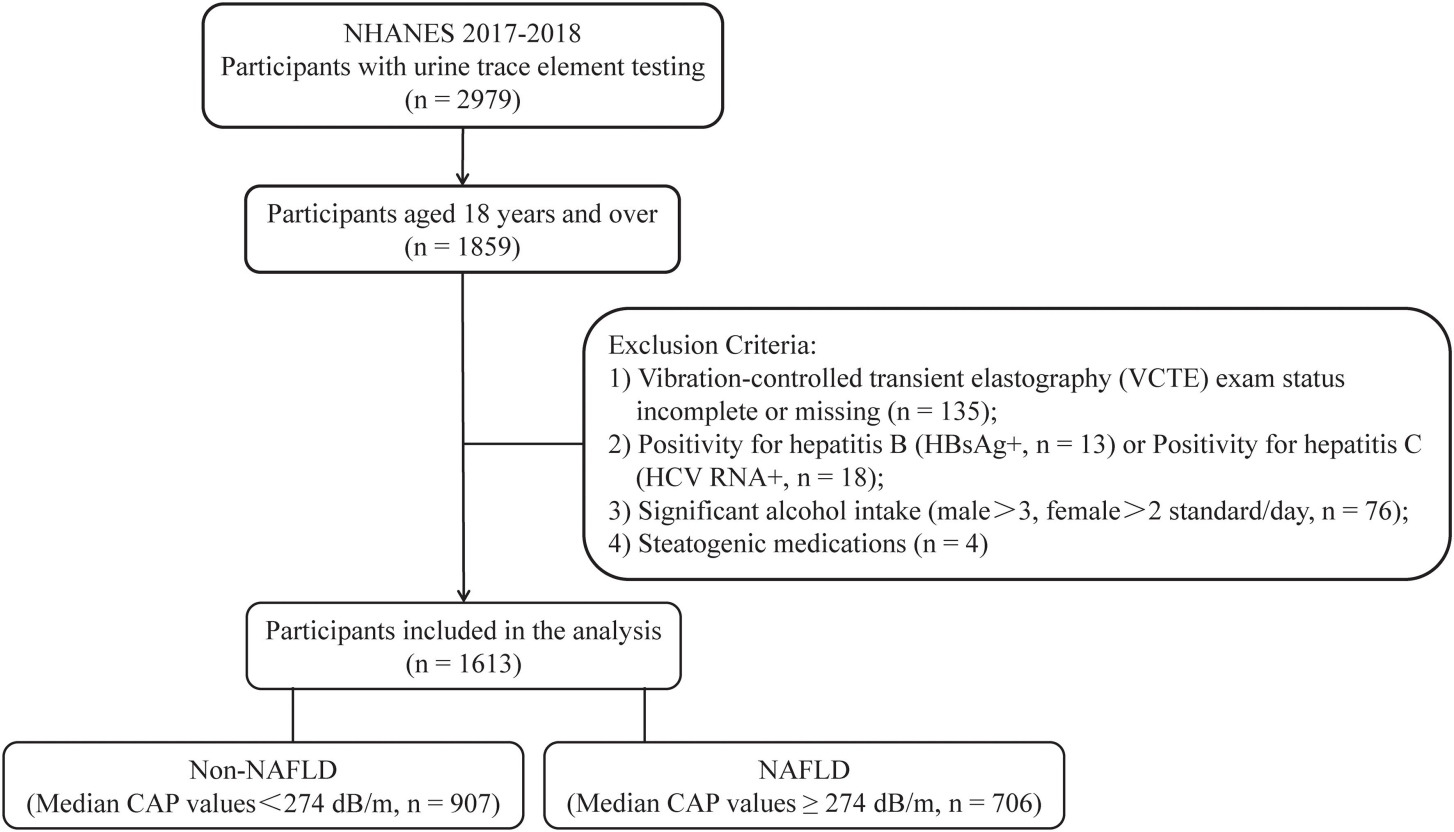
Process of choosing participants for the study. NHANES, National Health and Nutrition Examination Survey; HBsAg, Hepatitis B surface antigen; NAFLD, non-alcoholic fatty liver disease; and CAP, coefficient of attenuation parameter.

In this study, we utilized data from the NHANES 2017–2018 cycle, comprising 2979 individuals who underwent urine trace element testing. The study specifically focused on participants aged 18 years or above, totaling 1859 individuals. The presence of fatty liver tissue was determined by VCTE, with a median CAP value of >274 dB/m (sensitivity 90%, specificity 60%) (15), to identify liver steatosis. In this research, NAFLD was characterized as having a CAP value exceeding 274 dB/m in the absence of excessive alcohol consumption. **Figure 1** displays the criteria for the inclusion and exclusion of the study participants. Individuals who met any of the following criteria were not included in the study:1) insufficient or absent VCTE (n=135); 2) individuals who tested positive for Hepatitis B virus (HBV) surface antigen (n=13) or Hepatitis C virus (HCV) RNA (n=18); 3) individuals who consumed excessive amounts of alcohol (> 2 drinks/day for women and > 3 drinks/day for men, n=76) (16); 4) individuals who had been taking medications that affect fat metabolism for more than three months prior to their inclusion in the study (n = 4) (12). Ultimately, a total of 1613 participants were included in the analysis.

### 2.2 Analysis of quantities of elements in urine

During the NHANES, biological samples were gathered for laboratory testing, and biospecimens were collected at a mobile examination center, encompassing the gathering, processing, storage, and transportation of blood, urine, and various other specimen types. The collected urine samples were kept in suitable freezing conditions (2–4°C short-term and −20°C long-term) until the testing date. Comprehensive data regarding the testing outlined in the NHANES laboratory data can be found at https://wwwn.cdc.gov/nchs/nhanes/continuousnhanes. Here, Trace element contents were determined using inductively coupled plasma mass spectrometry (ICP-MS). The limits of detection for Arsenic (As), Chromium (Cr), Iodine (I), Mercury (Hg), Nickel (Ni), Barium (Ba), Cadmium (Cd), Cobalt (Co), Cesium (Cs), Molybdenum (Mo), Manganese (Mn), Lead (Pb), Antimony (Sb), Tin (Sn), Thallium (Tl), and Tungsten (W) were 0.230, 0.190, 2.400, 0.130, 0.310, 0.060, 0.036, 0.086, 0.023, 0.130, 0.800, 0.030, 0.022, 0.018, 0.090, and 0.018 micrograms per liter, respectively. Values below the detection limit were substituted with the square root of 2.

### 2.3 Covariates

Covariates such as demographic characteristics (age, gender, ethnicity, marital status, education level, and poverty to income ratio), anthropometric measurements, and body measurements (waist circumference, hip circumference, and body mass index (BMI)) were examined to identify potential confounders. Factors such as BMI, smoking habits, and physical activity, as well as existing medical conditions like high blood pressure, diabetes, and high cholesterol, along with laboratory measurements such as glycated hemoglobin, insulin levels, blood glucose, albumin levels, and levels of alanine transaminase, were taken into consideration.

The calculation of physical activity levels in the NHANES involved determining the metabolic equivalent (MET) and considering factors such as activity type, weekly frequency, and duration. This information was collected using a questionnaire. The formula used to calculate physical activity (MET-min/week) was MET multiplied by the weekly frequency and duration of each exercise (17), and a history of questionnaires or clinical criteria was used to diagnose hypertension, diabetes, and hyperlipidemia.

### 2.4 Statistical analysis

R programming software (https://www.r-project.org/, version 4.2.3) was utilized for statistical analysis and data calculations. Continuous variables were expressed as mean ± standard deviation, whereas numbers and percentages were used to express categorical variables. Trace element contents in urine displayed normal distribution and was represented by the median (M) and quartile. Comparison of categorical variables between groups was conducted using the chi-square test, while the independent t-test or Wilcoxon test was employed for comparison of continuous variables between groups. The Pearson correlation was utilized for examining the correlation between each trace element in urine, while the trend test was employed to analyze the levels of trace elements in urine. Trace element levels in urine were categorized into Quartiles 1–4 (Q1–Q4) groups based on quartiles, with the Q1 group serving as the benchmark group. To assess the relationship between urinary trace elements and NAFLD, we constructed both univariate and multivariable Logistic regression models. Odds ratio (OR), adjusted OR (aOR), and 95% Confidence intervals (CI) were calculated to analyze the results. Model A was modified to account for age, sex, race, educational level, and marital status. Additionally, Model B was adjusted to include BMI, smoking status, and physical activity, as these factors could potentially introduce bias. Considering the strong connection between liver steatosis and metabolism, Model C was modified to include hypertension, diabetes, and hyperlipidemia adjustments from model B. The relationship between urinary trace elements and NAFLD was modeled using a RCS approach to capture non-linearity. Considering the imbalanced distribution of urinary trace element levels, they were incorporated into the model following logarithmic (lg) conversion. To investigate the correlation between levels of trace elements in urine and the likelihood of developing NAFLD. Statistical significance was determined as P values less than 0.05 on both sides.

## 3 Results

### 3.1 Basic information

This study included a total of 1613 respondents after excluding participants who did not meet the requirements. Among them, 51.3% were female, with an average age of 49.87 years, and 58.9% were in a marital or cohabiting relationship; 14.8% were Mexican, 9.1% were Hispanic, 33.8% were non-Hispanic white, 23.1% were non-Hispanic black, and 19.2% belonged to diverse ethnic backgrounds. Moreover, 80.4% had high school education qualifications or higher, and 39.3% had middle income; in this particular study group, the occurrence rate of NAFLD was 43.77% (n = 706). Apart from variations in educational attainment and poverty/income ratio, the hypertension, diabetes, hyperlipidemia and other attributes displayed statistically significant disparities between the non-NAFLD and NAFLD groups (**Table 1**).

**Table 1:** Comparison of characteristics between NAFLD and non-NAFLD groups based on the 1613 subjects from NHANES 2017-2018.

Characteristics	Total (N=1613)	Non-NAFLD (n=907)	NAFLD (n=706)	*P*
Demographics Characteristics				
**Age (years)**	49.87 +/- 18.52	47.35 +/- 19.24	53.11 +/- 17.03	*< 0.001*
**Gender**				*< 0.001*
Male	786 (48.7)	402 (44.3)	384 (54.4)	
female	827 (51.3)	505 (55.7)	322 (45.6)	
**Ethnicity**				*< 0.001*
Mexican American	239 (14.8)	100 (11.0)	139 (19.7)	
Other Hispanic	147 (9.1)	84 (9.3)	63 (8.9)	
Non-Hispanic White	545 (33.8)	295 (32.5)	250 (35.4)	
Non-Hispanic Black	372 (23.1)	240 (26.5)	132 (18.7)	
Other Race	310 (19.2)	188 (20.7)	122 (17.3)	
**Marital Status**				*0.001*
Never married	894 (58.9)	466 (55.8)	428 (62.8)	
Married or living with a partner	354 (23.3)	195 (23.4)	159 (23.3)	
Widowed/Divorced/Separated	269 (17.7)	174 (20.8)	95 (13.9)	
**Education Level**				*0.526*
Less than 9th grade	316 (19.6)	172 (19.0)	144 (20.5)	
High school graduate	389 (24.2)	213 (23.5)	176 (25.0)	
Some college or AA degree	543 (33.7)	317 (35.0)	226 (32.1)	
College graduate or above	361 (22.4)	204 (22.5)	157 (22.3)	
**Poverty Income Ratio**				*0.542*
1.3 or less	412 (29.5)	239 (30.5)	173 (28.2)	
(1.3, 3.5)	549 (39.3)	299 (38.1)	250 (40.8)	
> 3.5	436 (31.2)	246 (31.4)	190 (31.0)	
Physical Examination and Lifestyle Characteristics				
**Smoking Status**				*0.028*
Never	971 (60.2)	557 (61.4)	414 (58.6)	
On smoking	257 (15.9)	155 (17.1)	102 (14.4)	
Quit smoking	385 (23.9)	195 (21.5)	190 (26.9)	
**BMI (Kg/m^2^)**				*< 0.001*
< 18.5	24 (1.5)	23 (2.6)	1 (0.1)	
18.5-24.9	413 (25.9)	354 (39.4)	59 (8.5)	
25-29.9 -	501 (31.4)	304 (33.9)	197 (28.2)	
30 or higher	658 (41.2)	217 (24.2)	441 (63.2)	
**Waist Circumference (cm)**	99.81 +/- 17.46	92.05 +/- 14.47	109.86 +/- 15.82	*< 0.001*
**Hip Circumference (cm)**	106.78 +/- 14.70	101.93 +/- 12.42	113.04 +/- 15.05	*< 0.001*
**Physical Activity (MET-min/w)**				*< 0.001*
< 600	612 (37.9)	310 (34.2)	302 (42.8)	
600-2999.	453 (28.1)	253 (27.9)	200 (28.3)	
P 3000	548 (34.0)	344 (37.9)	204 (28.9)	
Comorbidity				
**Hypertension**				*< 0.001*
NO	1013 (62.9)	623 (68.8)	390 (55.3)	
Yes	597 (37.1)	282 (31.2)	315 (44.7)	
**Diabetes**				*< 0.001*
NO	1329 (84.9)	810 (91.4)	519 (76.3)	
Yes	237 (15.1)	76 (8.6)	161 (23.7)	
**Hyperlipemia**				*< 0.001*
NO	1059 (65.9)	651 (71.9)	408 (58.0)	
Yes	549 (34.1)	254 (28.1)	295 (42.0)	
Laboratory Features				
**HbA1c (%)**	5.83 +/- 1.05	5.58 +/- 0.74	6.14 +/- 1.28	*< 0.001*
**Insulin (uU/ml)**	14.48 +/- 18.12	9.22 +/- 7.03	21.07 +/- 24.49	*< 0.001*
**Glucose (mg/dL)**	114.11 +/- 36.75	104.50 +/- 25.97	126.25 +/- 44.11	*< 0.001*
**Albumin (g/dL)**	4.07 +/- 0.32	4.09 +/- 0.33	4.04 +/- 0.32	*0.002*
**ALT (U/L)**	22.29 +/- 18.74	19.10 +/- 18.31	26.29 +/- 18.52	*< 0.001*
**AST (U/L)**	21.61 +/- 11.78	20.72 +/- 11.54	22.73 +/- 12.00	*0.001*
**ALP (IU/L)**	78.94 +/- 24.48	76.80 +/- 24.58	81.62 +/- 24.10	*< 0.001*
**GGT (IU/L)**	28.68 +/- 29.83	23.07 +/- 23.21	35.73 +/- 35.25	*< 0.001*
**Total Cholesterol (mg/dL)**	186.75 +/- 40.31	183.91 +/- 39.36	190.30 +/- 41.22	*0.002*
**Triglycerides (mg/dL)**	143.84 +/- 123.56	115.26 +/- 77.36	179.66 +/- 156.90	*< 0.001*

Continuous variables are expressed as the mean ± standard deviation and categorical variables as numbers and percentages. Nonalcoholic fatty liver disease; BMI, body mass index; HbA1c, glycohemoglobin; ALT, alanine transaminase; AST, aspartate aminotransferase; ALP, alkaline phosphatase; GGT, gamma-glutamyl transferase.

The p-value represents the difference between Non NAFLD and NAFLD.

### 3.2 Comparison of trace elements found in the urine of carious demographic groups

**Figure 2:**
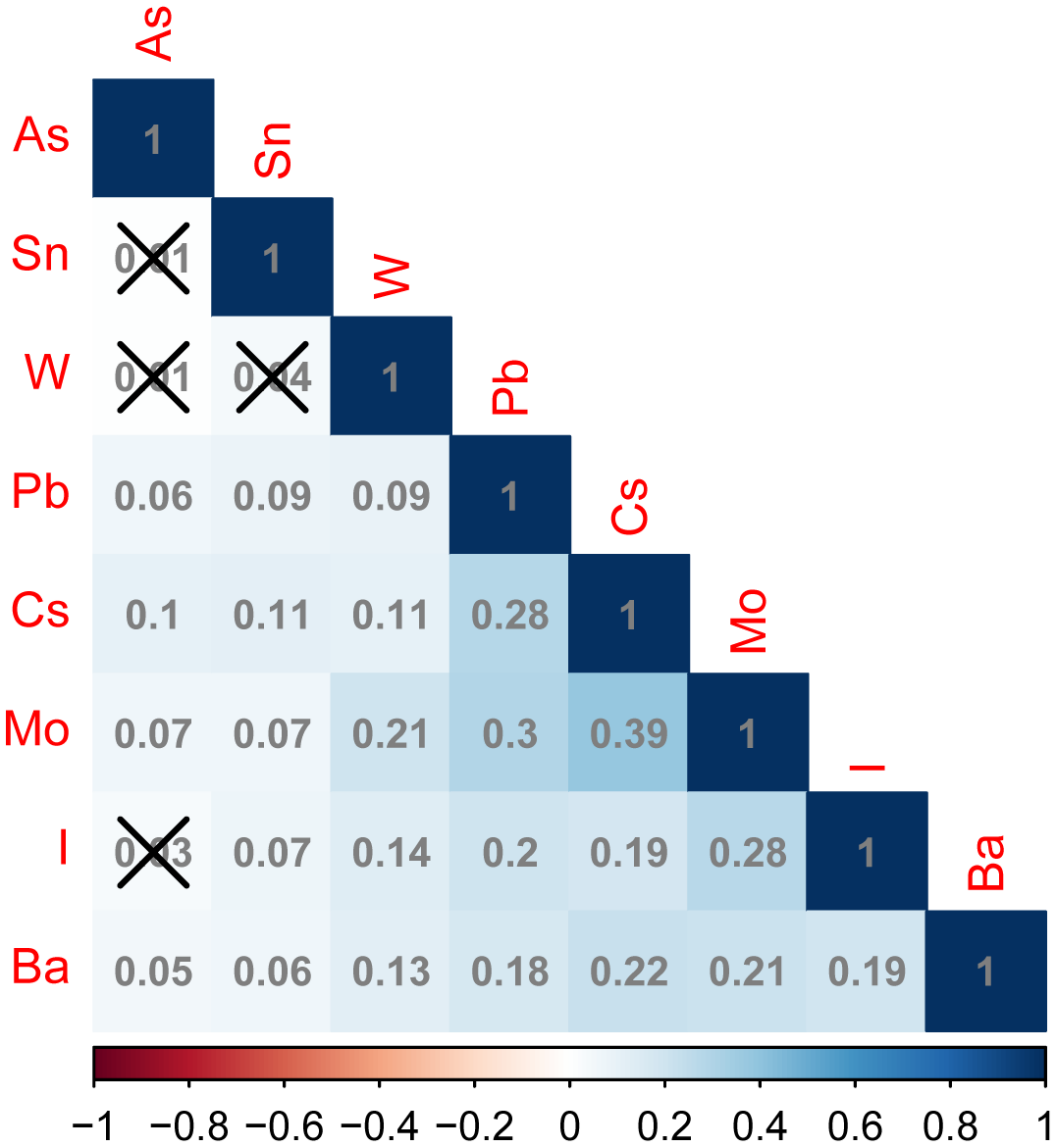
Correlation plots of different urinary trace elements between the non-NAFLD and NAFLD groups. In the correlation matrix, blue and red represent positive and negative correlations, respectively. Additionally, the intensity of the color indicates the strength of the correlation. The number in the box indicates the value of correlation coefficient between trace elements in urine, above the box “×” Indicates that this correlation coefficient value is not statistically significant (P > 0.05). arsenic (As), iodine (I), barium (Ba), cesium (Cs), molybdenum (Mo), Pb (lead), Sn (tin), and tungsten (W).

The levels of the 16 trace elements in the urine of the non-NAFLD and NAFLD groups are shown in **Table 2**. The NAFLD group exhibited significantly higher concentrations of eight trace elements, namely As, I, Ba, Cs, Mo, Pb, Sn, and W, than the those in the non-NAFLD group (P < 0.05). The median (interquartile range) concentrations of these eight trace elements in the NAFLD group were 6.99 (3.77–15.26), 125.65 (75.08–231.78), 1.04 (0.48–2.16), 4.86 (3.16–7.13), 38.71 (21.26–65.60), and 0.33, respectively (0.19–0.58), 0.49 (0.24–1.17), and 0.07 (0.03–0.11) *µ*g/l, respectively. The concentration distributions of the trace elements Cr, Hg, Ni, Cd, Co, Mn, Sb, and T did not show any significant differences between the two groups (P>0.05). **Figure 2** displays the correlation results between each trace element, obtained from the correlation analysis of the eight urine trace elements and the differences observed among groups. The eight trace elements exhibited a positive correlation (correlation coefficient >0); however, their correlation was weak (correlation coefficient < 0.4), and certain correlation coefficients did not reach statistical significance (P>0.05).

**Table 2:** Comparison of 16 trace elements in urine based on the NHANES 2017-2018 data of 1613 individuals.

Trace elements M (Q1-Q3) (ug/L)	Total (N=1613)	Non-NAFLD (n=907)	NAFLD (n=706)	*P*
**As**	6.45 (3.44-14.63)	6.22 (3.11 14.35)	6.99 (3.77-15.26)	*0.010*
**Cr**	0.13 (0.13-0.27)	0.13 (0.13-0.27)	0.13 (0.13-0.27)	*0.239*
**I**	117.30 (68.28-207.85)	110.45 (62.60 197.08)	125.65 (75.08-231.78)	*0.001*
**Hg**	0.13 (0.09-0.37)	0.09 (0.09-0.36)	0.15 (0.09-0.37)	*0.153*
**Ni**	1.18 (0.67-1.91)	1.16 (0.64-1.94)	1.21 (0.72-1.88)	*0.159*
**Ba**	0.96 (0.44-1.99)	0.87 (0.42-1.87)	1.04 (0.48-2.16)	*0.008*
**Cd**	0.21 (0.10-0.41)	0.20 (0.10-0.41)	0.22 (0.11-0.41)	*0.261*
**Co**	0.41 (0.25-0.66)	0.41 (0.24-0.66)	0.42 (0.25-0.65)	*0.577*
**Cs**	4.62 (2.72-6.82)	4.43 (2.51 6.52)	4.86 (3.16-7.13)	*< 0.001*
**Mo**	35.95 (19.01-63.21)	34.23 (16.63-60.99)	38.71 (21.26-65.60)	*0.005*
**Mn**	0.09 (0.09-0.15)	0.09 (0.09-0.15)	0.09 (0.09-0.16)	*0.137*
**Pb**	0.31 (0.17-0.55)	0.29 (0.16-0.53)	0.33 (0.19-0.58)	*0.008*
**Sb**	0.04 (0.03-0.08)	0.04 (0.02-0.08)	0.05 (0.03-0.07)	*0.229*
**Sn**	0.47 (0.22-1.09)	0.46 (0.20-1.03)	0.49 (0.24-1.17)	*0.048*
**Tl**	0.17 (0.10-0.26)	0.17 (0.09-0.25)	0.18 (0.11-0.26)	*0.136*
**W**	0.06 (0.03-0.11)	0.06 (0.03-0.11)	0.07 (0.03-0.11)	*0.036*

NAFLD, Nonalcoholic fatty liver disease; M, Median; As, Arsenic; Cr, chromium; I, Iodine; Hg, Mercury; Ni, Nickel; Ba, Barium; Cd, Cadmium; Co: cobalt; Cs, Cesium; Mo, Molybdenum; Mn, Manganese; Pb, Lead; Sb, Antimony; Sn, Tin; Tl, Thallium; W, Tungsten.

The p-value represents the difference between Non NAFLD and NAFLD.

### 3.3 Logistic regression analysis of the levels of eight urinary trace elements

**Figure 3:**
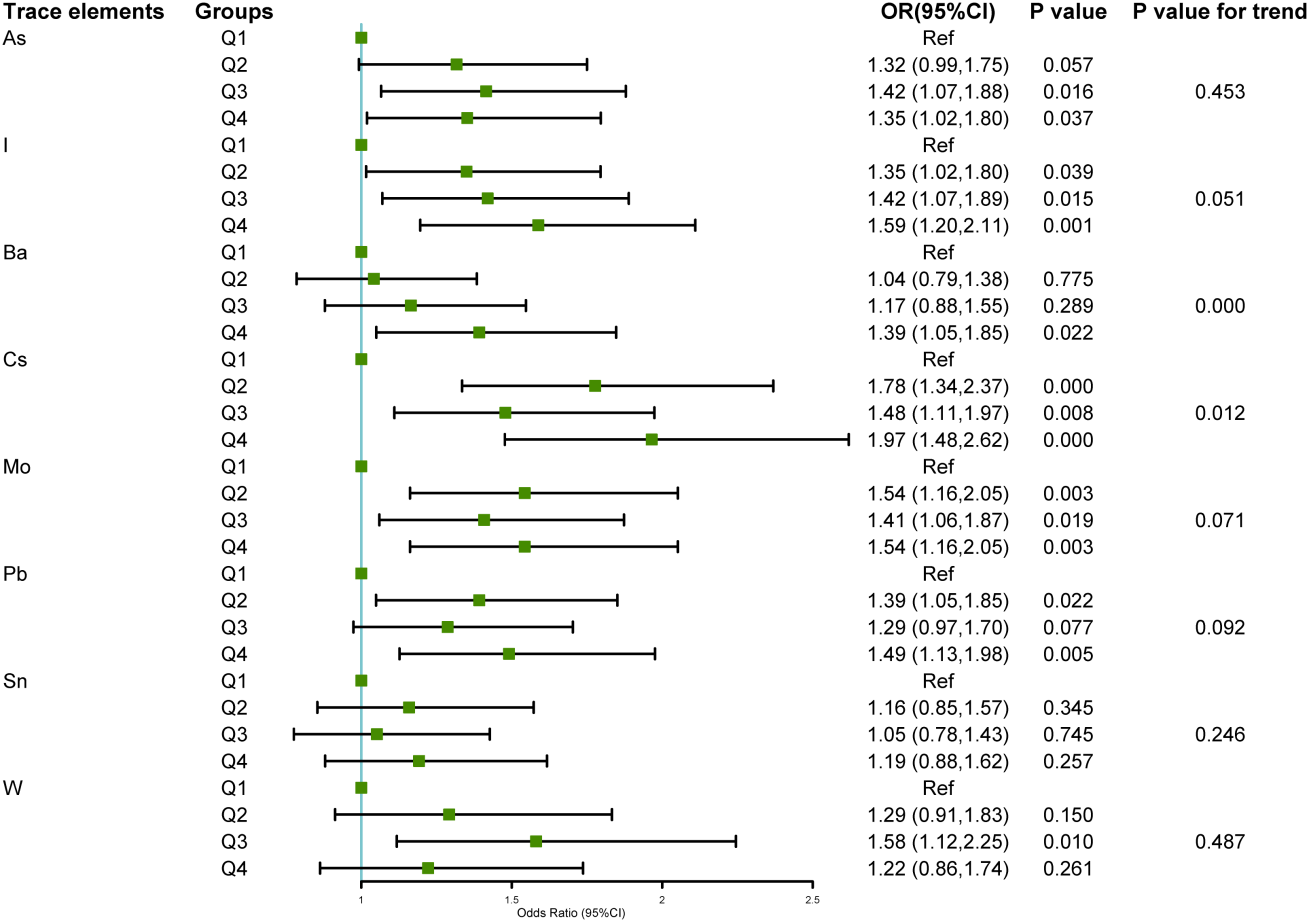
Relationship between the eight urinary trace elements in the NHANES 2017–2018 dataset and NAFLD.The association between urinary trace elements and NAFLD was examined using univariate logistic regression. The findings were displayed as forest plots, and the trace element levels were categorized based on quartiles, with the Q1 group serving as the baseline. A chi-square test for trend was employed. Odds ratio (OR), 95% Confidence interval (CI), Arsenic (As), Iodine (I), Barium (Ba), Cesium (Cs), Molybdenum (Mo), Lead (Pb), Tin (Sn), and Tungsten (W).

Univariate and multivariate logistic regression analyses were conducted using the quartile concentrations of As, I, Ba, Cs, Mo, Pb, Sn, and W in urine samples from various NAFLD groups, as independent variables, and the NAFLD group, as the dependent variable (non-NAFLD =0, NAFLD=1). The risk of NAFLD was higher with increases in urinary barium (chi-square trend = –3.48, P < 0.001) and cesium concentrations (chi-square trend = –2.26, P = 0.012), as depicted in **Figure 3**. Analysis of single variables showed that upon comparison of the Q1 of As, the risk of NAFLD increased in the Q2, Q3, and Q4 categories, with corresponding OR (95% CI) values of 1.32 (0.99, 1.75), 1.42 (1.07, 1.88), and 1.35 (1.02, 1.80), respectively. In comparison to that of the Q1 value of I, the likelihood of NAFLD increased in the Q2, Q3, and Q4 categories, with OR (95%CI) values of 1.35 (1.02, 1.80), 1.42 (1.07, 1.89), and 1.59 (1.20, 2.11), respectively. The risk of NAFLD in the Q4 group was 1.39 times higher compared to that in the Q1 group. The likelihood of NAFLD in the Q2, Q3, and Q4 categories of cesium increased by approximately 0.5–1 fold, compared with that in the Q1 category, with corresponding OR (95%CI) values of 1.78 (1.34, 2.37), 1.48 (1.11, 1.97), and 1.97 (1.48, 2.62), respectively. The likelihood of NAFLD in the molybdenum Q2, Q3, and Q4 categories increased by approximately 0.5-fold, compared with that in the molybdenum Q1 category. The corresponding OR (95%CI) values were 1.54 (1.16, 2.05), 1.41 (1.06, 1.87), and 1.54 (1.16, 2.05), respectively. In comparison to that in the lead Q1 group, the risk of NAFLD escalated in the lead Q2 and Q4 groups, with corresponding OR (95%CI) values of 1.39 (1.05, 1.85) and 1.49 (1.13, 1.98), respectively. In contrast to that in the tungsten Q1 group, the tungsten Q3 group exhibited a statistically significant increase in the likelihood of NAFLD, with an OR (95% confidence interval) of 1.58 (1.12, 2.25). No statistical correlation was found between the tin concentrations in urine and the risk of NAFLD.

To investigate the association between urinary trace elements and NAFLD, a multivariate logistic regression model was used to account for potential confounding variables. **Table 3** presents the outcomes of the three adjusted models. Compared with that of the Q1 group, the top quartile of Ba was associated with a higher likelihood of developing NAFLD (barium model A 1.61 [1.18, 2.20]; Model B 1.43 [1.01, 2.04]; Model C 1.52 [1.06, 2.18]). The risk of NAFLD was higher in the Q2 and Q4 groups, particularly with elevated Cs concentrations in Q2 to Q4. Additionally, the risk of NAFLD was elevated by approximately 0.5–1-fold, compared with that in the Q1 group. In Model A (adjusted for demographic characteristics), urinary I and Mo emerged as potential risk factors for NAFLD; however, they were no significant factors in Model B (adjusted for BMI, smoking status, and physical activity) and Model C (adjusted for hypertension, diabetes, and hyperlipidemia). After adjustment, the statistical correlations between urinary As, Pb, Sn, and W concentrations and NAFLD risk disappeared.

**Table 3:** Logistic regression analysis based on the levels of 8 trace elements in 1613 NAFLD and non-NAFLD groups from NHANES 2017-2018.

Trace elements	A Model		B Model		C Model	
	aOR (95%CI)	*P*	aOR (95%CI)	*P*	aOR (95%CI)	*P*
As						
Q1	1.00 (Ref)		1.00 (Ref)		1.00 (Ref)	
Q2	1.26 (0.93, 1.71)	*0.135*	1.13 (0.80, 1.60)	*0.480*	1.13 (0.79, 1.61)	*0.514*
Q3	1.31 (0.97, 1.78)	*0.078*	1.16 (0.83, 1.63)	*0.393*	1.14 (0.80, 1.62)	*0.468*
Q4	1.38 (1.02, 1.88)	*0.038*	1.38 (0.98, 1.95)	*0.069*	1.39 (0.97, 1.99)	*0.071*
I						
Q1	1.00 (Ref)		1.00 (Ref)		1.00 (Ref)	
Q2	1.37 (1.01, 1.86)	*0.040*	1.20 (0.85, 1.70)	*0.290*	1.22 (0.86, 1.74)	*0.261*
Q3	1.36 (1.01, 1.84)	*0.045*	1.30 (0.93, 1.83)	*0.128*	1.27 (0.90, 1.80)	*0.180*
Q4	1.43 (1.06, 1.94)	*0.021*	1.23 (0.87, 1.72)	*0.239*	1.21 (0.85, 1.71)	*0.293*
Ba						
Q1	1.00 (Ref)		1.00 (Ref)		1.00 (Ref)	
Q2	1.06 (0.79, 1.43)	*0.698*	0.96 (0.69, 1.34)	*0.816*	1.02 (0.72, 1.44)	*0.919*
Q3	1.26 (0.93, 1.71)	*0.138*	1.21 (0.85, 1.70)	*0.288*	1.35 (0.94, 1.93)	*0.102*
Q4	1.61 (1.18, 2.20)	*0.003*	1.43 (1.01, 2.04)	*0.043*	1.52 (1.06, 2.18)	*0.024*
Cs						
Q1	1.00 (Ref)		1.00 (Ref)		1.00 (Ref)	
Q2	1.68 (1.24, 2.28)	*0.001*	1.76 (1.25, 2.49)	*0.001*	1.70 (1.20, 2.43)	*0.003*
Q3	1.45 (1.07, 1.97)	*0.017*	1.35 (0.96, 1.91)	*0.089*	1.37 (0.96, 1.96)	*0.080*
Q4	2.09 (1.54, 2.84)	*< 0.001*	1.88 (1.33, 2.66)	*< 0.001*	1.81 (1.27, 2.58)	*0.001*
Mo						
Q1	1.00 (Ref)		1.00 (Ref)		1.00 (Ref)	
Q2	1.50 (1.11, 2.02)	*0.008*	1.30 (0.93, 1.82)	*0.129*	1.39 (0.98, 1.97)	*0.063*
Q3	1.48 (1.10, 2.01)	*0.011*	1.18 (0.84, 1.66)	*0.349*	1.10 (0.77, 1.56)	*0.598*
Q4	1.51 (1.12, 2.05)	*0.008*	1.33 (0.94, 1.87)	*0.107*	1.28 (0.90, 1.82)	*0.167*
Pb						
Q1	1.00 (Ref)		1.00 (Ref)		1.00 (Ref)	
Q2	1.33 (0.98, 1.81)	*0.068*	1.26 (0.89, 1.79)	*0.191*	1.24 (0.87, 1.77)	*0.229*
Q3	1.15 (0.85, 1.55)	*0.374*	1.23 (0.87, 1.72)	*0.245*	1.26 (0.89, 1.80)	*0.194*
Q4	1.14 (0.83, 1.55)	*0.417*	1.20 (0.85, 1.71)	*0.304*	1.29 (0.90, 1.85)	*0.165*
Sn						
Q1	1.00 (Ref)		1.00 (Ref)		1.00 (Ref)	
Q2	1.27 (0.92, 1.76)	*0.144*	1.17 (0.81, 1.70)	*0.395*	1.21 (0.83, 1.76)	*0.330*
Q3	1.07 (0.77, 1.48)	*0.697*	0.96 (0.66, 1.39)	*0.824*	1.01 (0.69, 1.48)	*0.967*
Q4	1.32 (0.95, 1.84)	*0.094*	1.02 (0.71, 1.49)	*0.901*	1.09 (0.74, 1.60)	*0.668*
W						
Q1	1.00 (Ref)		1.00 (Ref)		1.00 (Ref)	
Q2	1.25 (0.86, 1.82)	*0.237*	1.13 (0.75, 1.71)	*0.566*	1.23 (0.81, 1.89)	*0.333*
Q3	1.54 (1.06, 2.24)	*0.024*	1.29 (0.85, 1.97)	*0.237*	1.27 (0.83, 1.99)	*0.256*
Q4	1.28 (0.88, 1.87)	*0.198*	1.12 (0.74, 1.72)	*0.588*	1.07 (0.69, 1.65)	*0.774*

Adjusted covariates: Model A: age, sex, race, marital status, and education level; Model B: based on the A model with further adjustment for body mass index (BMI), smoking status, and physical activity; Model C: hypertension, diabetes, and hyperlipidemia were further adjusted on the basis of model B. aOR, adjusted odds ratio; 95% CI, 95% Confidence interval; Q, Quartile; As, Arsenic; I, Iodine; Ba, Barium; Cs, Cesium; Mo, Molybdenum; Pb, Lead; Sn, Tin; W, Tungsten.

The p-value represents the difference between Q2-Q4 and Q1.

### 3.4 Dose-dependent relationship between the levels of trace elements in urine and the development of NAFLD

**Figure 4:**
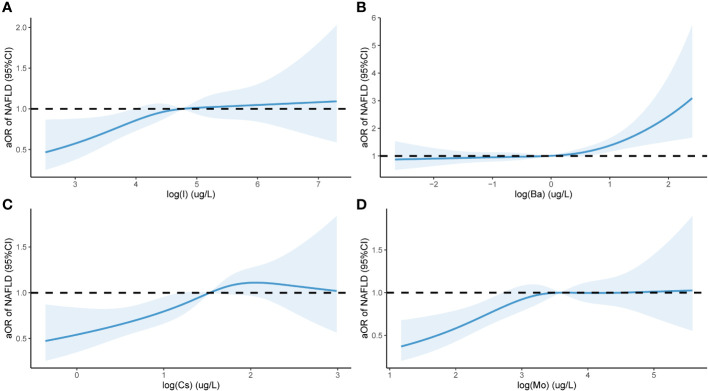
Urinary levels of eight trace elements and NAFLD in a population of 1613 subjects based on NHANES 2017-2018 Restricted cubic spline plot. aOR, adjusted odds ratio, adjusted odds ratio; 95% CI, 95% Confidence interval; NAFLD, Nonalcoholic fatty liver disease (NAFLD); I **(A)**; Ba **(B)**; Cs **(C)**; Mo **(D)**. The x-axis in the Figure 4 represents the log level of trace elements in urine, while the y-axis represents the adjusted ratio of NAFLD. The **Figure 4** is drawn using restricted cubic splines to show the nonlinear relationship between two variables.

Further investigations were conducted to explore the correlation between the risk of NAFLD and the concentrations of urinary trace elements (converted using logarithmic transformation) based on multivariate analysis. Analysis was conducted using the RCS model with four nodes. The findings, after adjusting the model for age, gender, race, education level, and marital status, and are displayed in **Figure 4**. An aOR value less than 1 indicates a reduced risk of NAFLD, whereas an aOR value greater than 1 indicates an elevated risk. **Figure 4A** displays a non-linear correlation between urinary I and the risk of NAFLD (χ=8.2, P=0.042), indicating a non-linear dose-dependent relationship. **Figure 4B** displays a non-linear correlation between urinary Ba and the risk of NAFLD (χ=19.39,2 P < 0.001, p- nonlinear =0.059). When the aOR value of NAFLD surpassed 1, the likelihood of NAFLD significantly increased with increasing urinary Ba concentration. **Figure 4C** displays a non-linear correlation (χ=14.36,2 p- nonlinear =0.003, P=0.341) between urinary Cs levels and the likelihood of NAFLD. **Figure 4D** displays a non-linear correlation (χ=12.88, 2 p- nonlinear =0.005, P=0.097) between the risk of NAFLD and urinary Mo. When the levels of urinary I, Cs, and Mo surpassed 1, the risk of NAFLD gradually increased.

## 4 Discussion

The study utilized health information from 1613 individuals residing in the United States to examine the correlation between trace elements found in urine and NAFLD. The primary findings were as follows: 1) there was a notable elevation in the levels of As, I, Ba, Cs, Mo, Pb, Sn, and W in the urine of individuals with NAFLD; 2) an increase in the levels of Ba and Cs in the urine corresponded with an increased risk of NAFLD. The risk of NAFLD was associated with elevated levels of Ba and Cs in urine, which were identified as independent risk factors. Additionally, a non-linear dose-dependent relationship was observed between urinary I, Mo, Ba, and Cs levels and the risk of NAFLD.

Most trace elements are absorbed by the body through the digestive tract, respiratory system and skin, transported in the blood, and exist in body fluids, cells and tissues. According to different ionic properties and dissolution characteristics, for example, cadmium is excreted in both urine and feces (18), the trace elements are excreted out of the body through different ways, such as the urinary system, digestive system, skin system, etc (19). The liver is the first line of natural defense against toxicity in the human body by filtering out and excreting harmful chemicals from the blood and detoxifying harmful chemicals through a variety of mechanisms; thus, there is an increased likelihood of the liver being affected by trace metal toxicity following exposure to toxins. Urine is one of the main ways to excrete waste. Previous studies have suggested the association between urinary trace elements and NAFLD.

In a previous study, a combination of metals in urine was positively associated with the occurrence of NAFLD, particularly in females. Among the metals, Ba and Cs were identified as the two most significant metals whose levels in urine were associated with the prevalence of NAFLD. These findings align with the results obtained in the current study. Nevertheless, the prevalence of NAFLD is influenced by various factors aside from gender, including BMI, smoking habits, level of physical activity, and the existence of complications like hypertension, diabetes, and hyperlipidemia. These additional factors have the potential to impact research outcomes, and research has indicated a strong correlation between metabolic dysfunction and physical activity in NAFLD (20). Consequently, in this study, we considered various factors, such as age, sex, ethnicity, level of education, marital status, BMI, smoking habits, physical activity, hypertension, diabetes, hyperlipidemia, and other disease-related factors. Confounding factors were identified through three models, and the association between levels of urinary Ba and Cs and NAFLD was further validated. Additionally, RCS analysis was employed to examine the dose-dependent relationship between the concentrations of urinary Ba and Cs and the onset of NAFLD. Specifically, we showed, for the first time, that the elevated levels of Ba and Cs in urine were significantly associated with the risk of NAFLD across the three adjustment models.

The impact of Ba content on NAFLD remains unclear; however, a study conducted in rural China discovered that elevated levels of plasma Ba were associated with an increased likelihood of central obesity (21). Moreover, excessive generation of free radicals may be linked to the mode of operation of Ba as the overproduction of ROS is facilitated by Ba, resulting in oxidative stress and ultimately causing functional alterations in the livers rats. These alterations impair mitochondrial function in hepatocytes near the portal vein, ultimately triggering apoptosis. Apoptosis disrupts the balance between lipid and protein oxygenation and redox states, potentially compromising the integrity of hepatocyte membranes. Consequently, hepatotoxicity occurs in rats (22). An alternative potential mechanism involves hormone levels; previous studies have indicated that Ba exhibits significant estrogenic activity (23). Furthermore, estrogen positively influences the differentiation of 3T3-L1 adipocytes and accumulation of lipids (24). This could explain the stronger association observed between urinary Ba levels and NAFLD in women (25).

A study conducted in Greece, which involved 189 participants, examined plasma trace elements during various stages of NAFLD and demonstrated a notable association between plasma Cs levels and the occurrence of NAFLD. However, the primary mechanism through which Cs operates remains unclear (26). After entering the human body, the levels of Cs in the kidneys, skeletal muscles, liver, and red blood cells increase (27). This could potentially contribute to liver damage, although the primary effect of Cs on the human body is its ability to obstruct K+ currents, leading to arrhythmia, QT prolongation, and torsade de pointes. These effects can result in systemic hypokalemia (28). However, the connection between this and the development of NAFLD does not appear to be significant. Metabolic factors like obesity and diabetes are closely correlated with the development of NAFLD. In a study conducted with middle-aged women in the United States, it was discovered that there was an inverse relationship between Cs levels and the likelihood of developing metabolic syndromes, determined by the environmental risk score system (29). However, when examining children, exposure to Cs during pregnancy was significantly associated with childhood obesity (30). An investigation using exposure omics techniques revealed a positive association between blood Cs levels resulting from early life environmental exposure and the occurrence of childhood obesity (31). Therefore, whether Cs exposure impacts lipid metabolism through embryonic or early childhood exposure and is associated with NAFLD, or whether Cs exposure participates in the occurrence of NAFLD through direct or indirect peroxidation stress processes, similar to other heavy metals and trace elements, requires further research.

Excessive iodine intake is associated with thyroid disease; however, research on the association between urinary iodine and NAFLD is lacking. However, some studies have suggested that excessive iodine consumption may disrupt the structure, metabolism, and function of cell membranes, potentially leading to liver, kidney, and pancreatic damage (32). This disruption could explain the non-linear dose-dependent relationship observed here between I levels and NAFLD. As for Mo, findings on its correlation with NAFLD are inconsistent, and several studies have indicated that the presence of Mo in the bloodstream is inversely related to the likelihood of NAFLD in male residents from China (33). This outcome is influenced by ethnicity, BMI, smoking habits, level of physical activity, and certain medical conditions.

This study provides certain contributions to existing research. First, for the diagnosis of NAFLD, CT, MRI and ultrasound can be used as imaging methods, but CT has ionizing radiation exposure, MRI is limited by high cost, and ultrasound is vulnerable to liver fibrosis and physical characteristics, while VCTE is recommended as a noninvasive detection method for the diagnosis of NAFLD (34, 35). The diagnostic standard for NAFLD was the CAP value measured by VCTE, which offers improved accuracy for diagnosis and eliminates the possibility of missed diagnoses due to the utilization of biochemical indicators like fat index. Furthermore, different from previous studies focusing on correlation, we used the restricted cubic spline models (RCS), to explore the dose effect relationship between urine trace element and NAFLD. Findings indicate that the effect of trace element in urine on NAFLD is not a simple linear increase or decrease, but a threshold effect, indicating the complexity of the interaction between trace element and liver disease, which fills in the blank of previous studies. Next, we employed three models for adjusting and evaluating the confounding variables, which encompassed factors closely associated with NAFLD, including duration of physical activity, presence of hypertension, diabetes, BMI, and others. Overall, we concluded that the presence of Ba and Cs in urine were the critical factors contributing to NAFLD.

However, this cross-sectional study has some limitations. Further investigations are required to examine the cause-and-effect connection between trace elements and the risk of NAFLD. Due to the unavailability of liver biopsy data in the NHANES dataset, the utilization of the VCTE examination method as a diagnostic criterion not only restricts the sample size that can be obtained from the NHANES database but also necessitates further investigation into potential residual confounding factors, including urine concentration, the duration and timing of trace element exposure and excretion pathway, in order to enhance the accuracy of relevant research.

## 5 Conclusion

In summary, this study further validated the association between urinary trace minerals and the likelihood of NAFLD in adults residing in the U.S. Elevated levels of Ba and Cs in urine showed a strong positive correlation with NAFLD. Furthermore, there was a non-linear dose-dependent relationship between the concentration of these elements and NAFLD, emphasizing the need for increased focus on their role in the prevention and treatment of this condition. Additional investigations are required to elucidate the biological mechanism linking Ba and Cs exposure to NAFLD, as well as to develop approaches to enhance or minimize exposure to associated trace elements.

## Data availability statement

The original contributions presented in the study are included in the article/supplementary material. Further inquiries can be directed to the corresponding author.

## Ethics statement

Publicly available datasets were analyzed in this study from NHANES. The studies involving humans were approved by the National Health Statistics Center’s Ethics Review Committee. The studies were conducted in accordance with the local legislation and institutional requirements. The participants provided their written informed consent to participate in this study.

## Author contributions

CW: Writing – original draft, Formal analysis. XS: Data curation, Writing – original draft. YF: Writing – review & editing, Conceptualization, Investigation. PG: Software, Visualization, Writing – original draft. PW: Writing – original draft, Validation. SY: Writing – review & editing, Supervision.
